# The Reimbursement Rate of New Rural Cooperative Medical Scheme and Self-Rated Health Among Rural Middle-Aged and Elderly

**DOI:** 10.3389/fpubh.2022.627169

**Published:** 2022-04-08

**Authors:** Xingquan Xie, Ying Hu

**Affiliations:** ^1^Department of Public Affairs Management, Chengdu University of Technology, Chengdu, China; ^2^West China Biomedical Big Data Center, West China Hospital, Sichuan University, Chengdu, China

**Keywords:** self-rated health, New Rural Cooperative Medical Scheme (NRCMS), family production theory, reimbursement rate, rural middle-aged and elderly

## Abstract

**Objectives:**

The ultimate goal of the New Rural Cooperative Medical Scheme (NRCMS) is to improve physical and psychological health and aim to provide equitable, affordable, cost-effective healthcare services for all rural people. One of our major concerns from the perspective of policy outcome is whether middle-aged and elderly can benefit from the insurance to improve self-rated health. The main objectives of this study are to answer the questions that the reimbursement rate of the NRCMS is a possible explanation of why and how rural middle-aged and elderly shift from non-medical service inputs to medical service to produce health based on a family production theory.

**Methods:**

Data were obtained from the China Health and Retirement Longitudinal Study (CHARLS) conducted in 2018, which involved 1,030 rural adults aged 45 years and older, and ordinal logistic regression estimator and two-step regression were used to examine these assumptions. Our approach controlled for the health status of those people at the same administrative level of the hospital.

**Results:**

Our study shows some interesting results. First, the reimbursement rate of NRCMS predicted a higher level of SRH among rural middle-aged and elderly, but that all of the indirect effect of it on SRH could be explained in total by satisfaction of local medical services utilization (ab = 0.0492). Second, the results further showed that the odds ratio of satisfaction from affordable, convenient, high-quality medical services is 2.402 times (*p* < 0.01) greater for those with higher reimbursement levels than for their counterparts with lower reimbursement. Third, the odds ratios of inpatient care visit, outpatient care visit, and physical examination among policyholders of NRCMS are also 1.116, 1.628, and 1.08 times greater, respectively, than their counterparts who are not satisfied with these local medical services.

**Conclusions:**

Our results concluded that generous insurance reimbursement can reduce the price of healthcare and costs of utilization that both had a dramatic effect on SRH among middle-aged and elderly when their demand for medical treatment is incurred. The government should focus on the healthcare cost, utilization, and health benefit calculations of health insurance policy options at the stage of rapid aging in rural China.

## Introduction

Rural China is facing an increasingly serious population aging problem. At the end of 2019, the population of elderly people aged 60 years and older increased to about 253.88 million, accounting for 18.1% of China's total population (1). Along with the aging population in rural China, the current health care system and public health insurance benefits for the aged are unable to meet all their needs for better health status. It is well-known that chronic diseases have been the main disease burden of the rural elderly in China. The death toll caused by chronic diseases has accounted for 87% of the total death toll in the country, and its disease burden accounts for about 70% of the total disease burden in China (2). Chronic diseases represent a serious health hazard to rural middle-aged and elderly' physical and psychological health and affect their quality of life, health adjusted life expectancy, and perceived health because of the high prevalence of morbidity, healthcare costs, more treatment cycles, and a high mortality rate. The chronic disease problems among rural older people has rapidly become a major public health challenge in rural China. Improving access to healthcare services for rural middle-aged and elderly is one of the principal objectives of public health insurance. Because rural older adults are often disadvantaged by illness and poverty, enhanced attention on this disadvantaged population is critical to health equity and eliminating poverty (3).

To solve the problem of poor health status and the majority of rural residents who remained uninsured, China launched the New Rural Cooperative Medical Scheme (NRCMS) in 2003 (4). At its inception, the NRCMS was designed to provide public health coverage to most rural residents to improve utilization of health services, prevent rural households from incurring catastrophic health expenditures (CHE), and promote their good health (5). The 2002 State Council Policy Document No. 13 stipulates that the New Rural Cooperative Medical Scheme is a public health insurance system for most rural residents who still lack health insurance (6). Most scholars also refer to the New Rural Cooperative Medical Scheme as 'the NRCMS (7), which contains detailed guidelines for the implementation of rural public health insurance for each participant and manager to follow (8). Moreover, the NRCMS is a premium-based plan financed by a combination of government funds and individual users, with the Chinese government subsidizing 70 to 80% of the insurance premium to overcome poverty and improve access to healthcare services due to rising prices for the insured (9). The reimbursement and generosity of the NRCMS vary by the level of medical facilities: (1) The reimbursement rate for hospitalization expenses is about 90% if the insured visited a village clinic or township hospital or community healthcare center for medical treatment. (2) The reimbursement rate for hospitalization expenses is from 70 to 80% if the insured visited the second-class hospital or county-level hospitals for medical treatment. (3) The reimbursement rate for hospitalization expenses is 50 to 60% if the insured visited tertiary hospitals or downtown hospitals in metropolitan districts for medical treatment (10). Although participation in the NRCMS is voluntary, the NRCMS also requires full household participation or surrender, with either none or all of their folks participating in the social health insurance scheme to reduce adverse selection (11). Whether one has good health or bad health, participation in NRCMS to improve access to healthcare services is one of the main motivations for enrollees.

A key issue in the recent debate over preventing chronic disease in China is whether the extensive coverage of insured individuals by the NRCMS promotes their health (11). Some studies provide evidence that the NRCMS within familiar contexts made a tremendous impact on producing health and improving equity in access to healthcare utilization (12). It is acknowledged that the reimbursement rate is higher for primary health facilities and lowest for city-center hospitals such as tertiary hospitals in order to influence rural middle-aged and elderly to stay close to home and go to their local healthcare centers (13). However, there is the main concern that the rural and other second-tier facilities are viewed by the public as being very low quality, which is not conducive to producing health for middle-aged and elderly in rural China (14). Thus, the generous reimbursement rate of NRCMS aims to modify consumer behavior, encouraging patients to seek medically appropriate levels of care at the appropriate facility, and dissuade them from going to the city-center tertiary hospital simply out of perception that it is always best. There are a few researchers who explored the relationships between social health insurance and self-rated health (SRH) based on specific age groups, and their results showed that social health insurance is strongly associated with health status in different age groups (15). Some studies pointed out that perceived health is strongly influenced by institutional factors such as the healthcare system, healthcare utilization, social health insurance (16). It is universally acknowledged that the ultimate goal of the NRCMS is to improve physical and psychological health for all rural people and is aimed at the provision of equitable, affordable, cost-effective healthcare services at the stage of healthy aging. One of our major concerns from a perspective of implementation effect is whether middle-aged and elderly can benefit from NRCMS in terms of improved access to healthcare services (17).

Thus, many studies have found that NRCMS is closely associated with individual health status through healthcare utilization because it can eliminate the gap in access to health care services and the financial burden of disease in vulnerable groups (18). However, few studies employed healthcare utilization in the analysis of the mediation effect of NRCMS on SRH for middle-aged and elderly. The main aims of this paper were to examine the mediating effects of the satisfaction from the cost, convenience, and quality of local medical services utilization on the relationship between the reimbursement rate for NRCMS and self-rated health among rural middle-aged and elderly. Insights derived from our research may not only contribute directly to our understanding of the healthcare cost, utilization, and health benefit calculations of health insurance policy options at the stage of rapid aging in China, but also explain how the reimbursement rate of NRCMS impacts SRH by the satisfaction from the cost, convenience and quality of the local healthcare system in rural middle-aged and elderly.

## Theoretical Arguments and Framework

### Family Production Theory and Healthcare Utilization

In this paper, we aim to examine how the NRCMS's reimbursement rate impacts SRH, applying the Becker family health production model as the theoretical framework (19). Although Mushkin (20), Becker (21), and Fuchs (22) have concluded that an individual's health can be viewed as one form of human capital, no one has researched the influence factor of health production. The traditional economic theory demonstrated that health is the output of human desire, and demand for healthcare is the inevitable outcome of family production (23). People often use a healthy diet, more physical exercise, positive genetic and environmental factors, and cost-effective medical treatment to produce health. Classical demand theory assumed that those hygiene factors above are deemed services and goods purchased in the health market enter consumers' utility function (24). In this approach, the family produces health with the purchase of these market goods such as exercise in the gym, good nutrition from food, more rest time at the expense of working time, etc (25). Some economists have emphasized that family members choose among these market goods based on their relative prices which can include time prices and currency prices. If one good becomes cheaper, we will marginally shift from other more expensive goods to cheaper goods. The “transfer” is marginal-we will use more healthcare service, but less exercise in gym, etc. for instance. Since the most fundamental demand curve is downward-sloping in economics (26), the quantity and quality of the supply of healthcare are usually negatively associated with the market price of healthcare. Bates et al. predicted that the underlying health production function allows for subsidies for medical prices in these markets to alter the optimal amount of health and also stimulate the great demand for cheaper medical service, measured, say, by insurance reimbursement (27). Under this condition, individuals would rather shift from expensive exercise, diet, recreation, leisure or traveling, etc., to utilize cheap healthcare. Social health insurance can reduce healthcare prices compared with other government subsidies; then, lead to a large increase in the quantity of healthcare demand among rural middle-aged and elderly.

Family health production theory states that preventive services are the best input into the production of health but generous health insurance can lower the out-of-pocket price of curative inputs relative to the price of preventive inputs and thereby distort the choice of inputs. We can conclude from those certain conditions above, rising insurance reimbursement rates may simultaneously bring the out-of-pocket medical price down and then increase the quantity of medical care demand. Thus, the insured has much less input of healthy lifestyle into the production of health. The conclusion is consistent with Ehrlich and Becker to a certain extent that the healthy people covered by generous health insurance are often not producing health in the best ways such as going to the gym, early detection of diseases or taking preventive care (28). However, the ex-ante moral hazard effect in healthcare is mostly theoretical because health insurance does not entirely cover the utility loss related to illness (disamenity, pain, disability) because the beneficiary of health insurance still takes the risk of illness and death any time anywhere.

Therefore, the public health insurance among the household may remain attractive because the purchase price of social health insurance is less than the other health inputs, and there is a higher probability of curing illness completely through healthcare utilization. Grossman found that although people inherit an initial stock of health that may be increased by health input, that initial stock depreciates over-time at an increasing rate (29). He asserted that illness or health need, measured by the level of the rate of depreciation that rises with age, definitely be positively associated with medical services utilization (29). Geriatric diseases and biological factors related to aging raise the price of maintaining health and cause the poor and uninsured rural older residents with critical illness to possibly shortened expected lifespan until death is forced to be “chosen”. It is a common phenomenon that older people have higher demands for the production of health and desire to purchase health insurance because their stock of health depreciates over-time at an increasing rate. The aging population is a major driver of the rapid annual growth in national health spending and in the demand for healthcare because the overall health among middle-aged and elderly declines with the probability of sickness on the rise. As healthcare consumption in middle-aged and elderly increases, their desire to buy affordable, equal, cost-effective, convenient insurance is grows (30). It has been suggested that health insurance influences the efficiency of the production process of health because it reduces the price of medical care at the point of being sick in the future, so rural older people would like to choose lower premiums but higher reimbursement of insurance. The NRCMS that covers rural residents is their only and best choice. This is really the law of demand (31)- the lower the out-of-pocket medical price, the more people will be willing and able to purchase healthcare services such as inpatient care, outpatient care, physical examinations, etc. Every rural inpatient who is a beneficiary of NRCMS will receive great insurance gains as the government reimburses them for 50% to 90% of total medical costs of hospitalization. Compared to many private medical insurances in China, the New Rural Cooperative Medical Insurance is “a good bargain” for rural older people. If the policyholders of NRCMS fall ill unexpectedly, those people prefer to produce health through insured medical treatment rather than using more expensive inputs, such as exercise, nutrition, etc.

**Figure 1:**
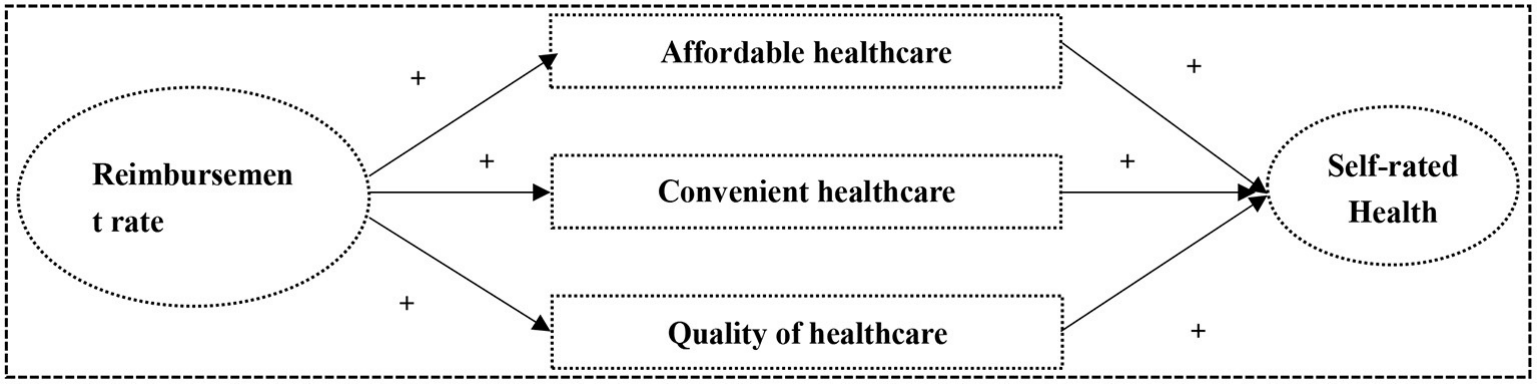
The NRCMS' reimbursement rate impacts the self-rated health among rural middle aged and elderly people through local medical service utilization.

Our research is different from that of others. This study assumed that the reimbursement rate of NRCMS (RRN) impacts SRH among rural middle-aged and elderly through the satisfaction of affordable, convenient, and high-quality medical services based on family production theory as the underlying framework (Figure 1). First, the NRCMS' generous reimbursement can reduce the relative price of healthcare when medical treatment is incurred, so the enrollees would rather choose more affordable medical care to produce health than other inputs when they fall sick. Within the family production framework for examining the effect of a health input on health, gross input in health capital is produced by household production functions whose initial direct inputs also depend on certain “institutional variables” (32). Social health insurance has a positive effect on healthcare cost and utilization. It should be realized in this framework that generous health insurance reduces out-of-pocket medical expenses; so, the insured prefer to shift from nutrition, exercise, recreation, etc. to medical care for producing health. The theory supported the view that generous reimbursement has significantly increased the likelihood of healthcare utilization, such as inpatient care, outpatient care, physical examination, etc. Second, the NRCMS is targeted for improving the accessibility of a medical facility where the insured will get to these medical facilities quickly and conveniently to prepare for producing health in time. Family production theory states that patients will consider spatial factors in examining accessibility to healthcare services (33). Since distance costs are part of the total medical costs among rural middle-aged and elderly, local medical facilities should remove the geographic barrier between the healthcare provider and patients, and those input shifts rely on the efficiency of spatial access (34). The NRCMS aims to provide convenient healthcare and a simple reimbursement approval process, which attracts a lot of middle-aged and elderly to receive medical treatment faster for producing health (35). Last but not least, the anticipated reimbursement rate of NRCMS is lower for the higher-level inpatient medical facilities. This will encourage the local primary medical facilities to provide high-quality medical service as soon as possible to increase profits in the long run. It is common knowledge that local governments and local medical facilities to make every effort to provide comprehensive and high-quality medical services to attract thousands of residents to visit for medical care nearby in order to increase profits or fiscal revenue. So, in that sense, the generous reimbursement rate of NRCMS is positively correlated with the quality of local medical service and then good self-rated health. However, in the context of Chinese medical systems where simpler health needs are handled in the rural local medical facilities and more complex cases are triaged up to the more tertiary hospitals. Thus, in our statistical modeling, we will use health status, administrative level of hospitals and type of medical facility as covariate variables to control the medical appropriateness of going to the city-center tertiary hospital simply out of health need among rural middle-aged and elderly.

Although some studies found that the NRCMS implementation failed to reduce total and out-of-pocket (OOP) medical costs and improve access to healthcare for low-income groups such as rural middle aged and elderly people (36), other studies have found that NRCMS have had a positive impact on reducing healthcare costs and improving access to healthcare among enrollees (37). The effects of NRCMS on healthcare utilization and production of health might be heterogeneous among existing literature since these studies did not demonstrate to the primary beneficiary how to choose among these inputs based on their relative prices. One of the reasons for this is that previous studies would rather use NRCMS' coverage variable than employ insurance reimbursement rates, however, insurance coverage alone cannot comprehensively reflect and measure healthcare costs and utilization. Moreover, these key independent variables are limited measures of insurance claims and do not reflect educating those primary beneficiaries on how to benefit from the NRCMS reimbursement and how much insurance compensation the insured specifically receives from the insurance. Therefore, the independent variable of interest for our study is the reimbursement rate of NRCMS. It is a better substitute for insurance coverage or participation. This study also aimed to examine the assumption that the positive relationship between NRCMS' reimbursement rate and SRH is mediated by the satisfaction from low-cost, convenient, high-quality local medical service utilization among rural middle-aged and elderly.

## Materials and Methods

### Data Sources

This article is a retrospective study based on the China Health and Retirement Longitudinal Study (CHARLS) in 2018 to explore the relationship between the reimbursement rate of NRCMS and SRH in a national sample of rural adults aged 45 years and older. CHARLS is surveyed by the Institute of Social Science Survey of Peking University in China, with the purpose of reflecting a large-scale national, biennial household, and representative survey of China's health, retirement, and population (sampling protocol is publicly available at http://charls.pku.edu.cn/index/en.html). The inclusion criteria of the participants in our analysis were: (1) rural household registration and; (2) aged 45 years and older and;(3) enrolled in NRCMS or Urban and Rural Resident Medical Insurance (URMI) and; (4) no missing value in all variables. This study removed those samples with missing values because the samples of the CHARLS are large enough. Ultimately, 1,030 respondents were selected, with 837 and 193 in the NRCMS group and URMI group, respectively. All the respondents in the CHARLS provided informed written consent that was in accordance with the Declaration of Helsinki. This paper is a retrospective study based on CHARLS, in which the Ethical Review Committee of Peking University provided ethical approval of the survey (Approval number: IRB00001052-11015).

### Variable Definition and Measures

#### Self-Rated Health

This study adopted the concept of health by WHO as “a state of complete physical, mental and social well-being, and not merely the absence of disease and infirmity” (38). So, the dependent variable of SRH is employed in our article to reflect the outcome of producing health. The dependent variable is self-rated health that was measured by the following question, “Would you say your health is very poor, poor, fair, good or very good?” A five-item scale tapped answers to the question in the CHARLS. This item category scored with 5 points: “1 = very poor”, “2 = poor,” “3 = fair”,“4 = good”,“5 = very good”.

The reimbursement rate of NRCMS (RRN) represents the place where subjects received healthcare services and it is the key independent variable in our analysis. Two items were used to represent the concept of the reimbursement rate in CHARLS. The two items include total medical costs of hospitalization and out-of-pocket costs in CHARLS. First, total medical costs of hospitalization only include fees paid to the hospital, including ward fees but excluding wages paid to a hired nurse, transportation costs, and accommodation costs for yourself or family members. Second, the “out-of-pocket” part was investigated by this question “how much will you pay out of pocket for the total medical costs of hospitalization, after reimbursement from insurance?” Third, we know that total medical costs of hospitalization minus out-of-pocket medical costs equal the reimbursement for medical costs of hospitalization. Thus, RRN = [1- (out-of-pocket medical costs of hospitalization / total medical costs of hospitalization)], which scores on the RRN were coded from 0 to 1.

It should be noted that the mediating variable is adopted in this study to answer the question, “how does RRN impact SRH in rural middle aged and elderly people?”. The mediating variable is scored with 5 points is the utilization of local medical services, are you satisfied with the cost, convenience and quality of local medical services when you visited local medical facilities? This item was scored as follows: 1 signified “not at all satisfied”, 5 signified “completely satisfied”. Three binary variables—whether you have been hospitalized in the past year, whether you visited the medical facility for outpatient care last month, and whether you had a physical examination since the last interview—are employed to reflect healthcare utilization incurred.

Our analysis also controlled for a set of factors that may affect respondents' health conditions and access to healthcare. These included variables for individual social-economic demographics, health status, type and level of health facilities among the respondents, for social-economic demographics, including age, gender, education, marital status, etc. The correct identification of our statistic model that relates an outcome y (SRH) to an independent variable (RRN) relies on the assumption that there is the same or approximate health status among middle aged and elderly people in our sample. Because the scores of activities of daily living (ADL), numbers of disabilities, numbers of chronic conditions, depression, and lifestyle may be proxies for current health status, these sets of covariate variables were controlled in our study. Furthermore, in the context of Chinese medical systems, simpler health needs are handled in the rural local medical facilities and more complex cases are triaged up to the more tertiary hospitals. An obvious concern in our context is critical illness, since patients who received less reimbursement are those who went to the downtown tertiary hospital. Some of these may be more severe cases, and therefore may have reported worse health status. Thus, the second series of binary covariate variables in our study measure whether the subject incurred critical illness, such as stroke, cancer, heart disease, emphysema, etc. In addition, we also controlled the level of this medical facility and the type of this medical facility which patients visited, so, the object of our study is the rural older patients who visited for last medical care at the same type and level of health service facility.

### Methodology

**Figure 2:**
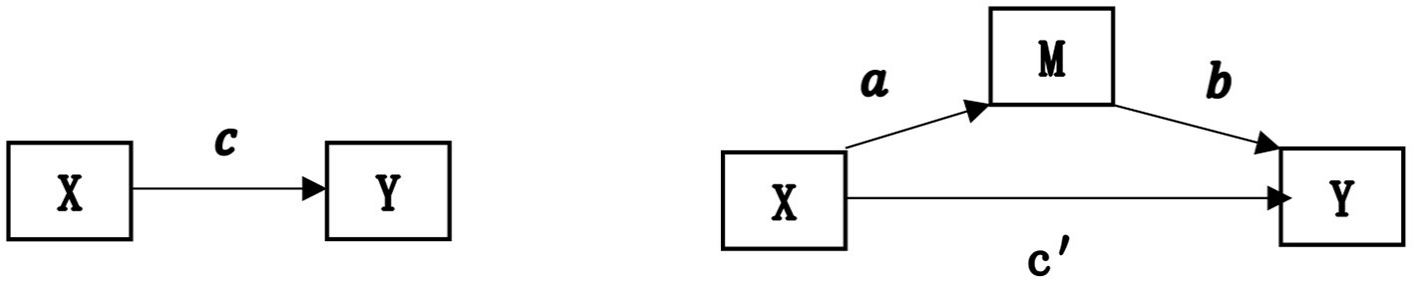
Influence path of the mediating variables. It illustrates that the independent variable X affects the dependent variable Y through the mediating variable M. X is the reimbursement ratio of the New Rural Cooperative Medical Scheme (RRN). Y represents self-rated health. M represents a “local medical service utilization”.

Because NRCMS, characteristic of local medical service, healthcare utilization, and SRH are usually discrete variables that are not applicable to stepwise regression referred by Baron and Kenny (39). The two-step logistic regression method that was developed by Zhao et al. (40) who present a nontechnical tutorial in hope of remedying a major defect of the step-by-step procedure method and Sobel z-test proposed by Baron and Kenny (39), to more accurately calculate and robustly test for the mediation effect (40). So, the two-step logistic regression is a good substitute for stepwise regression. In this paper, the statistical process by which RRN affects SRH through local medical service characteristics and healthcare utilization, as shown in Figure 2, can be divided into two phases.

Two equations below illustrate that the independent variable X affects the dependent variable Y through the mediating variable M. X is RRN,Y represents SRH, and M represents local medical service utilization and main characteristics (affordable, convenient, and high-quality). The product of regression coefficients a^*^b represents the indirect impact of RRN on SRH. Two-step logistic regressions estimate the parameters a and b, used to test the indirect effect (mediation effect). In addition, Tofighi et al. (41) recommended that the estimate of indirect effect *u*.*a*×*u*.*b*+σ*ab*; u.a is the mean of a, u.y is the mean of b (41).

*M* = *aX*+ε_1_ The first step (Ordinal logistic regression)

$Y=c^{{\;}^{\prime }}X+bM+\varepsilon_{2}$ The second step (Ordinal logistic regression)

In a nonrecursive three-variable causal model above, Zhao et al. (40) identified two patterns consistent with non-mediation and three with mediation: (1) No-effect non-mediation: Neither indirect effect nor direct effect exists. (2) Direct-only non-mediation: No indirect effect but only direct effect exists. (3) Indirect-only mediation: No direct effect but only mediated effect exists. (4) Competitive mediation: Direct effect and mediated effect both exist and the two point in opposite directions. (5) Complementary mediation: direct effect and indirect effect both exist, and the two point at the same direction (40).

## Results

Table 1 shows a descriptive summary of covariates and major variables categorized by insurance group. We reported the mean and standard deviation of each continuous variable, as well as the observations of each categorical variable. Table 1 shows the descriptive characteristics of the population of rural middle-aged and elderly. In 2018, the average age of rural middle-aged and elderly was approximately 60.51 ± 9.05 years. Table 2 is the results of two-way tabulations along with common Pearson's chi-squared that measures of association between different healthcare utilization and satisfaction of local medical services. The results indicated that satisfaction of local medical services are positively correlated with inpatient care, outpatient care and physical examination. Our study used two-step logistic regression to examine the effect of reimbursement of NRCMS on self-rated health in rural middle-aged and elderly.

**Table 1:** Descriptive analysis in rural aged 45 years and older, presented as mean / median, SD, N.

**Variables**	**NRCMS**			**URRMI**		
	***N***	**Mean/Median**	**SD**	***N***	**Mean/Median**	**SD**
SRH	837	3.086	0.937	193	2.842	1.021
RRN	837	0.456	0.252	193	0.62	0.286
Medservices	837	3.064	0.997	193	3.375	1.154
Age	837	60.51	9.054	193	59.851	8.370
Male	837	1	0.492	193	1	0.500
Mastatus	837	1	1.315	193	2.097	1.415
Education	837	3.787	1.305	193	2.226	1.190
Sleep	837	1	0.467	193	1	0.488
Smoke	837	0	0.312	193	1	0.246
Drink	837	0	0.438	193	0	0.422
Exercises	837	1	0.285	193	1	0.344
Physical examination	837	1	0.465	193	1	0.499
Soactivities	837	1.704	1.157	193	1.189	0.526
Diseases	837	1.942	1.173	193	1.761	1.043
Disability	837	1	0.417	193	1	0.497
Depression	837	6.367	5.727	193	9.839	6.900
ADL	837	1.791	0.603	193	1.93	0.644
Inpatient care	837	0	0.434	193	1	0.410
Outpatient care	837	1	0.386	193	1	0.375
Hospital type	837	3	0.665	193	4	0.904
Hospital level	837	1	0.793	193	2	0.592
Stroke	837	0	0.268	193	0	0.257
Cancer	837	0	0.145	193	0	0.110
HD	837	0	0.347	193	0	0.284
Emphysema	837	0	0.230	193	0	0.253

*RRN, reimbursement rate of NRCMS; SRH, Self-rated Health; Medservices, Cost, Convenience and Quality of Local Medical Services; Mastatus, Marital Status; Soactivities, Numbers of Social Activities; Diseases, Number of Chronic Diseases; ADL, Activities of Daily Living; HD, Heart disease*.

**Table 2:** Chi-squared test for independence of satisfaction of local medical services and healthcare utilization.

**Local medical services**	**Inpatient visit (*n*)**			**Outpatient visit (*n*)**			**Physical examination (*n*)**		
	**No**	**Yes**	**All**	**No**	**Yes**	**All**	**No**	**Yes**	**All**
Very dissatisfied	75	19	94	75	19	94	54	39	94
Somewhat dissatisfied	60	15	75	59	16	75	43	32	75
Neutral	383	76	459	379	80	459	259	200	459
Somewhat satisfied	192	43	235	192	43	235	117	118	235
Very satisfied	136	31	167	141	27	167	82	86	167
Chi^2^ test	Pearsonchi^2^(4) = 9.428		*P*-value = 0.049	Pearsonchi^2^(4) = 12.657		*P*-value = 0.013	Pearsonchi^2^(4) = 47.462		*P*-value = 0.000

As shown in Table 3, healthy middle aged and elderly people differed significantly from unhealthy middle aged and elderly people in health characteristics and self-rated health. With adjustment by control variables, such as the same health conditions, the same type and level of health service facility visited for medical care, these health characteristics were equally distributed in our ordinal logistic regression between the poor health group and the good health group. So, after controlling for those covariates above, the reimbursement of NRCMS is positively correlated with SRH in rural middle-aged and elderly The odds ratio of RRN is positive but not significant (1.331, p>0.05), indicating that the self-rated health reported by some rural middle aged and elderly people with the same health status receiving higher insurance reimbursement may be better than those with less insurance reimbursement. The results held true when our analysis accounted for the beneficiaries' overall health status, type and level of the medical facility they last visited.

**Table 3:** The RRN is associated with SRH in rural middle-aged and elderly.

**Model**	**Model 1**	**Model 2**	**Model 3**	**Model 4**	**Model 5**	**Model 6**
**Variables**	**SRH**	**Medservices**	**SRH**	**Inpatient care**	**Outpatient care**	**Physical examination**
Medservices			1.260^***^	1.116^***^	1.628^**^	1.080^***^
			(0.099)	(0.041)	(0.207)	(0.029)
RRN	1.331	2.402^***^	1.209	1.546^**^	2.041^***^	1.337
	(0.438)	(0.788)	(0.405)	(0.327)	(0.423)	(0.282)
Age	1.032^***^	1.023^**^	1.029^***^	1.029^***^	0.984^***^	1.060^***^
	(0.010)	(0.010)	(0.011)	(0.005)	(0.005)	(0.004)
Male	0.874	0.745	0.921	1.145	0.725^***^	0.796^***^
	(0.175)	(0.148)	(0.187)	(0.108)	(0.066)	(0.054)
Mastatus (Ref:Married)						
Separation	1.246	1.702	1.242	0.661^**^	0.893	0.982
	(0.502)	(0.666)	(0.504)	(0.121)	(0.127)	(0.105)
Divorce	0.944	2.846	0.789	1.519	1.226	0.773
	(0.838)	(2.978)	(0.693)	(0.755)	(0.598)	(0.326)
Single	0.598^**^	0.828	0.614^**^	1.050	1.112	0.926
	(0.135)	(0.184)	(0.139)	(0.109)	(0.114)	(0.072)
Education (Ref: Illiterate)						
Primary school	0.706	1.024	0.693	1.038	0.939	1.211^**^
	(0.186)	(0.269)	(0.184)	(0.126)	(0.110)	(0.107)
Middle school	0.603^**^	0.717	0.606^*^	1.270^*^	1.072	1.498^***^
	(0.155)	(0.183)	(0.157)	(0.156)	(0.127)	(0.135)
High school	0.432^***^	0.576^*^	0.459^***^	1.296^*^	1.231^*^	1.404^***^
	(0.125)	(0.169)	(0.134)	(0.178)	(0.155)	(0.135)
Associate degree	0.601	0.486^*^	0.622	1.266	1.364^*^	2.309^***^
	(0.243)	(0.191)	(0.254)	(0.247)	(0.235)	(0.299)
Bachelor's degree and Above	1.752	0.407	1.923	2.480	3.508^**^	4.221^***^
	(2.232)	(0.422)	(2.458)	(1.623)	(1.791)	(2.165)
Soactivities	0.956	0.956	0.965	1.002	1.178^***^	1.221^***^
	(0.115)	(0.113)	(0.117)	(0.060)	(0.057)	(0.047)
Sleep	1.037	1.233	1.033	0.984	1.126	1.287^***^
	(0.176)	(0.209)	(0.176)	(0.082)	(0.088)	(0.075)
Drink	0.902	1.115	0.899	1.397^***^	1.063	0.969
	(0.202)	(0.248)	(0.202)	(0.150)	(0.107)	(0.069)
Smoke	0.850^**^	0.567^**^	1.165	1.072	0.846	0.166^***^
	(0.054)	(0.148)	(0.209)	(0.082)	(0.129)	(0.065)
Exercises	1.574	0.982	1.629^*^	1.007	1.243	1.300^**^
	(0.447)	(0.277)	(0.465)	(0.141)	(0.184)	(0.139)
Diseases	0.768	0.936	0.765	1.846^***^	1.546^***^	1.440^***^
	(0.148)	(0.184)	(0.148)	(0.172)	(0.132)	(0.094)
Disability	0.740^*^	1.020	0.745^*^	1.102	0.979	1.086
	(0.120)	(0.178)	(0.121)	(0.092)	(0.083)	(0.075)
Depression	0.904^***^	0.964^***^	0.909^***^	1.016^**^	1.010	0.984^***^
	(0.012)	(0.012)	(0.012)	(0.007)	(0.006)	(0.005)
ADL	0.538^***^	0.903	0.542^***^	1.223^***^	1.288^***^	0.921
	(0.078)	(0.128)	(0.079)	(0.091)	(0.090)	(0.051)
Stroke	0.552^*^	0.675	0.578^*^	1.539^***^	0.979	1.290^*^
	(0.168)	(0.203)	(0.176)	(0.250)	(0.176)	(0.196)
Cancer	0.596	0.772	0.602	2.833^***^	1.199	1.168
	(0.234)	(0.297)	(0.237)	(0.813)	(0.377)	(0.327)
HD	0.649	0.687	0.639	0.928	0.797	1.445^***^
	(0.196)	(0.204)	(0.194)	(0.146)	(0.127)	(0.187)
Emphysema	0.897	1.072	0.874	1.779^***^	1.638^***^	1.189
	(0.230)	(0.279)	(0.226)	(0.244)	(0.224)	(0.149)
Hospital level (Ref: County/District hospital)						
Regional/City hospital	0.815	0.791	0.854	1.449^***^	1.247^***^	0.998
	(0.179)	(0.173)	(0.190)	(0.130)	(0.107)	(0.063)
Provincial/Affiliated to a ministry hospital	1.234	0.860	1.331	2.127^***^	0.944	0.571^***^
	(0.378)	(0.271)	(0.409)	(0.151)	(0.118)	(0.074)
Military hospital	0.822	1.401^*^	0.804	1.184^*^	0.922	0.769^**^
	(0.153)	(0.266)	(0.150)	(0.104)	(0.076)	(0.088)
Hospital type (Ref: Village clinic/Private clinic)						
Health care post	0.942	0.982	1.080^**^	1.006	1.058	0.716
	(0.100)	(0.043)	(0.036)	(0.031)	(0.152)	(0.165)
Township hospital	0.825	1.299	1.543^***^	0.681	1.051	0.998
	(0.186)	(0.273)	(0.225)	(0.853)	(0.045)	(0.081)
Community healthcare center	0.943	0.131^***^	1.745	0.653^**^	1.352^**^	0.876
	(0.583)	(0.060)	(0.718)	(0.137)	(0.199)	(0.188)
Specialized/Chinese medicine hospital	0.660^***^	0.902	0.979	1.425^***^	0.594^***^	1.429^*^
	(0.080)	(0.114)	(0.085)	(0.187)	(0.090)	(0.304)
General hospital	0.731^*^	0.797^**^	1.100	1.254^**^	1.051	0.998
	(0.126)	(0.071)	(0.066)	(0.114)	(0.045)	(0.081)
Constant				0.011^***^	0.212^***^	0.008^***^
				(0.005)	(0.090)	(0.003)
Observations	1,030	1,030	1,030	1,030	1,030	1,030
Pseudo R-squared	0.102	0.0440	0.109	0.109	0.0613	0.0642

*RRN, reimbursement rate of NRCMS; SRH, Self-rated Health; Medservices, Cost, Convenience and Quality of Local Medical Services; Mastatus, Marital Status; Soactivities, Numbers of Social Activities; Diseases, Number of Chronic Diseases; ADL, Activities of Daily Living; HD, Heart Diseases. Robust standard errors in parentheses.*

*** *p < 0.01,*

** *p < 0.05,*

* *p < 0.1*.

The analysis in the theoretical framework stresses that the RRN and SRH usually are not directly connected, but are indirectly related through healthcare utilization. Our results are consistent with most of the studies that lower medical cost was associated with the probability of healthcare utilization and with a higher level of perceived health among the rural older enrollees. As shown in Table 3, in a sense, it appears that a high reimbursement rate is positively correlated with local medical service utilization. In rural China, there was significant difference in utilization of local medical services between the low and high reimbursement groups after controlling for their health status, and both level and type of medical facility. Specifically, the results further showed that if a primary beneficiary of NRCMS had higher reimbursement, the odds ratio of the satisfaction from affordable, convenient, and high-quality local medical services utilization was 2.402 times (*p* < 0.01) greater than their counterparts with lower reimbursement. Furthermore, model 4 to model 6 in Table 3 showed that the odds ratio of inpatient visit, outpatient visit, and physical examination are 1.116, 1.628, and 1.08 times greater, respectively, for rural middle-aged and elderly who are satisfied with local medical service than those unsatisfied with it, controlling for their health status. This finding provides circumstantial evidence that the reimbursement of NRCMS is positively correlated with SRH in rural middle-aged and elderly.

These results supported the family production theory that the generous insurance reimbursement reduces the price of medical care at the point of being ill, so the insured would rather use more medical care than utilize nonmedical services to produce health, when controlling for health status. We discovered that the increase in RRN increased the probability of reporting their good health among rural middle aged and elderly people by controlling health status and health outcomes. In family theory, the quantity of health demanded often declines as healthcare prices rise. This result is consistent with the experience that generous health insurance reduces out-of-pocket expenses for medical care; then, rural middle aged and elderly people covered by generous NRCMS prefer to shift from expensive non-medical services to cheaper medical care when curing diseases is a top priority. In rural China, the probabilities of reporting good health are 1.26 times (*p* < 0.01)greater for middle-aged and elderly who are satisfied with local medical service than those unsatisfied with it, regardless of health status.

Table 4 shows specific indirect effects of the RRN on SRH that is mediated by the satisfaction of affordable, convenient, high-quality local medical services. Our results further found that the RRN predicted a higher level of SRH within these respondents, but that much of the indirect effect of RRN on SRH could be explained in total by satisfaction of cost, convenient, and high-quality local medical services (ab = 0.0492). We generated Bootstrap results for indirect effects from Stata software, and the 95% bias-corrected, accelerated confidence interval (BCa) is 0.0117 to 0.1304. These mediations are significant, because the BCa does not include zero (42). Although the direct effect of the RRN on SRH was about 0.1041, its BCa is from −0.2242 to 0.4255. That BCa obviously includes zero, and is therefore not significant. Therefore, the type of mediation in our study is indirect-only. As expected, our data support the hypothesized mediation story and extended it to family production theory that the RRN affects a distal dependent variable SRH through the satisfaction from local medical service utilization among rural middle-aged and elderly

**Table 4:** Build BCa and test mediation hypotheses using the bootstrap method.

**RRN > LMS > SRH**	**Coef**	**Bias**	**SE**	**95% BCa**	**Significant**
Indirect effect	0.0492	0.0011	0.0285	0.011727 0.130436	Yes
**RRN > SRH**
Direct effect	0.1041	0.0027	0.1605	−0.2242029 0.4254853	NO

*BCa, Bias-corrected and Accelerated Bias corrected Bootstrap*.

## Discussion

This article is the first to use a nationally representative survey targeting middle aged and elderly people in rural China to explore the relationship between NRCMS' reimbursement rate and SRH based on family production theory. Their mediation effect and health policy implications are discussed here. Our study found that the enrollees would like to use more medical care treatment to produce health when they fall sick, since the NRCMS' generous reimbursement can lower the price of healthcare, improve accessibility and quality of healthcare. It revealed that the reimbursement rate of NRCMS may be viewed as an external economic incentive for rural middle aged and elderly people to produce health by healthcare utilization and health system function. Our results suggested that healthcare costs and utilization had a dramatic and direct effect on better SRH among middle aged and elderly people when their demand for medical treatment is incurred.

Our explanation adopted the family production theory and extends it. Traditional family production theory suggested that rational man marginally shifts from more expensive inputs to cheaper inputs to maintain health. Our findings supported the hypothesis that middle aged and elderly people covered by generous health insurance would rather use more healthcare than non-medical services to produce health when medical treatment is incurred. We demonstrated that the reimbursement rate of NRCMS not only has a significant effect on healthcare utilization but also optimizes local medical facility functions and the degree to which healthcare services for primary beneficiaries increase the likelihood of desired health outcomes. These findings showed that the higher level of reimbursement rate of NRCMS is positively correlated with low-cost, convenient, and high-quality of local medical services that is expected to lead to a large increase in local healthcare utilization to improve self-rated health among rural middle-aged and elderly So, the main determinant of choice of those health inputs is whether the reimbursement rate of NRCMS raises or lowers their relative prices when demand for medical treatment is incurred among rural middle-aged and elderly. The rapidly increasing geriatric population is not only a major driver in the demand for national health spending and healthcare utilization, but also is susceptible to healthcare costs and their perceived health in rural China. Our study further found that the reimbursement rate of NRCMS not only impacts the employee's choice of health input, but also encourages local medical facilities to provide affordable, accessible, and high-quality healthcare services for those primary beneficiaries. In other words, only generous health insurance has a positive effect on the choice of health input among enrollees or else the utility of going to the gym and running on a treadmill exceeds the utility of going to the hospital and undergoing medical treatment. These findings revealed that a higher reimbursement rate of NRCMS is the external effective institutional economic factor that influences the choices of health input among enrollees. Our research extends family production theory from consumers' utility functions in health economics to social welfare analysis in health policy evaluation. This study has realized that institutional incentives for NRCMS and individuals' rational choices are equally important, and they are closely connected.

These findings provide further scientific evidence for the positive effect of NRCMS on healthcare utilization among middle aged and elderly people in rural China. Our results showed that the odds of middle aged and elderly people who are satisfied with local medical services utilizing inpatient care, outpatient care, and having physical examinations are one to two times greater, respectively, than their counterparts. Our result was consistent with the conclusions of some studies that NRCMS in rural China is the main health financing mechanism to secure access to adequate healthcare service for the insured at an equal, affordable, convenient price to produce health (43). However, our result differs from other studies in that we chose to use reimbursement rates than NRCMS coverage or enrollment indicators. The former option being superior, because it can reflect how those primary beneficiaries benefit from the NRCMS reimbursement and how much insurance compensation the insured specifically receives from the insurance benefit package. There has been much dispute over the question of the policy effect of NRCMS on healthcare costs, utilization and health status based on the differences in data sources, measurement, methodological differences in subject recruitment, and sample bias (44). These differences in age groups may have contributed to the different findings since older adults have a higher propensity for poor health, low incomes, the absence of generous health insurance, under-utilization of high-quality healthcare at city-center tertiary hospitals (45). Furthermore, our analysis also controlled for a set of socio-economic factors that may have impacted access and costs of healthcare utilization among rural middle aged and elderly people, such as health status, age, types of medical facilities, administrative level of medical facilities, etc. Thus, our study might better represent the true association between reimbursement rate of NRCMS and SRH among middle aged and elderly people in rural China than do studies using self-report data on healthy or younger groups without controlling for those demographic and health characteristics.

## Conclusion

Our study had several limitations. First, since our study draws data for analysis from the cross-sectional 2018 CHARLS, we must be cautious about inferring causal relationships. Secondly, although SRH acts as a health status proxy variable that appears both reproducible and reliable, this variable may also be underestimated or overestimated, because the respondents are often not truly aware of their real physiological and mental conditions. Third, although an individual's perceived health as the dependent variable in our analysis reflects the output from health service utilization, in a sense, the main concern is the degree to which SRH also impacts the person's choice of where they receive their medical care.

Our findings have far-reaching policy implications for promoting equality of NRCMS. First, our results suggest that the next public health reform in rural China should replace Fee-for-Service (FFS) with Diagnosis Related Group Systems (DRGs)-based reimbursement to achieve dual goals: cost containment and maximization of health output. Second, the reimbursement rate of hospitalization expenses should be increased in middle aged and elderly people at a disadvantage of health and economic status after the NRCMS merged with URMI came into effect. Third, the government should devote its attention to providing more affordable, cost-effective, convenient, and equitable health insurance for middle aged and elderly people who would like to use more medical services to produce health when their medical treatment is incurred in rural China. Last but not least, the government not only needs to focus on the NRCMS' design, implementation and reform, but also emphasize the fact that the health care costs and utilization are the vital nexus between the NRCMS and SRH that is a very important policy mechanism to produce health in a public health system. How to reduce the burden of disease and produce better health for rural middle aged and elderly people by bringing the NRCMS benefits of the package is one of the key priorities of rural China's public health insurance reform at the stage of rapid aging of the population.

## Data Availability Statement

The dataset supporting the conclusions of this article is available in the http://charls.pku.edu.cn/pages/data/2018-charls-wave4/en.html.

## Ethics Statement

All the respondents in the CHARLS provided informed written consent that was in accordance with the Declaration of Helsinki. This paper is a retrospective study based on CHARLS, in which the Ethical Review Committee of Peking University provided ethical approval of the survey (Approval number:IRB00001052-11015).

## Author Contributions

XX conceived, designed, and conducted the original research, who acquired and analyzed the data. Drafting the work or revised it critically about nearly 70% was a substantial contribution to XX. YH contributed to the methods, collection and interpretation of data, and proofreading of final paper together. Both authors contributed to the article and approved the submitted version.

## Funding

This study was funded by General Projects of the National Social Science Fund of China from 2018 to 2021(Approval Number: 18BZZ101).

## Conflict of Interest

YH was employed by West China Hospital, Sichuan University. The remaining author declares that the research was conducted in the absence of any commercial or financial relationships that could be construed as a potential conflict of interest.

## Publisher's Note

All claims expressed in this article are solely those of the authors and do not necessarily represent those of their affiliated organizations, or those of the publisher, the editors and the reviewers. Any product that may be evaluated in this article, or claim that may be made by its manufacturer, is not guaranteed or endorsed by the publisher.

## Abbreviations

RRN: reimbursement rate of NRCMS
SRH: Self-rated Health
RRN: reimbursement rate of NRCMS
Medservices, Cost: Convenience and Quality of Local Medical Services
Mastatus: Marital Status
Soactivities: Numbers of Social Activities
Diseases: Number of Chronic Diseases
ADL: Activities of Daily Living
HD: Heart disease
emphysema: Chronic Obstructive Pulmonary Disease
Bca: Bias-Corrected and Accelerated Confidence Interval.
